# Highly Fluorinated Methacrylates for Optical 3D Printing of Microfluidic Devices

**DOI:** 10.3390/mi9030115

**Published:** 2018-03-08

**Authors:** Frederik Kotz, Patrick Risch, Dorothea Helmer, Bastian E. Rapp

**Affiliations:** Institute of Microstructure Technology, Karlsruhe Institute of Technology, Hermann-von-Helmholtz-Platz 1, 76344 Eggenstein-Leopoldshafen, Germany; Frederik.Kotz@kit.edu (F.K.); Patrick.Risch@kit.edu (P.R.); Dorothea.Helmer@kit.edu (D.H.)

**Keywords:** 3D printing, perfluoropolyether, additive manufacturing, microfluidics, stereolithography

## Abstract

Highly fluorinated perfluoropolyether (PFPE) methacrylates are of great interest for transparent and chemically resistant microfluidic chips. However, so far only a few examples of material formulations for three-dimensional (3D) printing of these polymers have been demonstrated. In this paper we show that microfluidic chips can be printed using these highly fluorinated polymers by 3D stereolithography printing. We developed photocurable resin formulations that can be printed in commercial benchtop stereolithography printers. We demonstrate that the developed formulations can be printed with minimal cross-sectional area of 600 µm for monolithic embedded microfluidic channels and 200 µm for open structures. The printed and polymerized PFPE methacrylates show a good transmittance above 70% at wavelengths between 520–900 nm and a high chemical resistance when being exposed to organic solvents. Microfluidic mixers were printed to demonstrate the great variability of different designs that can be printed using stereolithography.

## 1. Introduction

Three-dimensional (3D) printing is of high interest for microfluidics due to the multitude of channel systems that need to be tested in the course of lab-on-a-chip device development. With 3D printing, prototypes and devices can be produced in a fast and effective manner. While many different techniques exist for 3D printing of microfluidic devices [1,2,3], for example, fused deposition modeling (FDM) [4], inkjet printing [5], two-photon printing [6], or stereolithography. While two-photon printing has been used to fabricate optical submicron structures [7,8], stereolithography remains the method of choice for most laboratories to fabricate microfluidic chips as it combines affordable machinery with high resolution [9]. Microfluidics started out with chips made from silicon and glass, but today polymers (thermosets, elastomers) are the preferred material class because polymer processing and structuring is considerably easier when compared to glass and silicon*.* One of the main perspectives of microfluidics is automation and miniaturization in biological as well as in chemical devices. While alternative materials, such as glass [10,11], are extremely chemically resistant, polymers are still the preferred choice for rapid fabrication as they do not require specialized fabrication equipment [12,13]. However, most polymers that are used for printing and fabrication of microfluidic chips are not stable when exposed to even low concentrations of chemicals, especially organic solvents. This is why there has been increasing interest in recent years in developing resistant materials, which could pave the way to simple fabrication techniques of solvent-resistant microfluidic chips. Concerning durability and chemical resistance, fluoropolymers stand out specifically. Fluorination affords the lowest known surface energies: the difluoromethylene (-CF_2_-) group, the difluoromethyl group (-CF_2_H), and the trifluoromethyl group (-CF_3_) possess surface energies of ~18 mN/m, ~15 mN/m, and ~6 mN/m, respectively [14]. For comparison, methyl groups (-CH_3_) have surface energies of ~23 mN/m [14]. In addition, the carbon/fluorine bond is the shortest bond in organic chemistry which makes fluorinated polymers outstandingly stable also in direct contact with organic solvents. There is a high interest in using fluoropolymers for microfluidic chips, and many applications have been reported. Commercial fluorinated thermoplasts such as Viton and Dyneon can be structured by hot embossing [15,16], or selectively bonded to parts of the chip that require chemical stability [17]. Commercially available perfluoropolyether (PFPE) polymers, like SIFEL, have been used for chip fabrication via, e.g., casting [18] or spin-coating [19]. Another strategy is to employ chemical vapor deposition techniques to deposit fluoropolymer films on microfluidic devices [20]. Photolithographic direct structuring of highly fluorinated polymers has been first reported using PFPE acrylates [21], which are now widely used for the fabrication of microfluidic devices [22,23]. Despite the interesting features of fluoropolymers, few attempts have been reported to structure fluoropolymers via 3D printing or for the fabrication of microfluidic devices. Recently, a method to fabricate pillar arrays (100 µm diameter, 400 µm height, 50 µm layer thickness, 150–200 µm spacing) by printing a custom-synthesized PFPE tetraacrylate was reported [24]. Here, we present a simple method for 3D printing of PFPE dimethacrylates for the fabrication of microfluidic devices. To the best of our knowledge, this is the first technique for 3D printing of microfluidic chips in highly fluorinated polymers.

## 2. Materials and Methods

*Materials:* All of the chemicals were used as received and were not purified any further. PFPE Fluorolink MD700 was purchased from Acota, Shrewsbury, UK. Acetone, 2-propanol, methanol (MeOH), dichloromethane (DCM), dimethylformamide (DMF), tetrahydrofuran (THF), toluene, and *n*-heptane were purchased from Merck, Darmstadt, Germany. Diphenyl(2,4,6-trimethylbenzoyl)phosphine oxide (TPO), phenylbis(2,4,6-trimethylbenzoyl)phosphine oxide (PPO) was purchased from Sigma Aldrich, Taufkirchen, Germany. Tinuvin 384-2 (T384-2) and Tinuvin 326 (T326) were kindly provided by BASF, Ludwigshafen, Germany. Sudan Orange G (SOG) was purchased from Sigma Aldrich. Elastosil RT 601 A/B was purchased from Wacker, Munich, Germany. Formlabs Tough was purchased from Formlabs, Somerville, MA, USA. The microfluidic channels were filled with a black, blue, and yellow printing ink that was purchased from ESM online, Hirschberg, Germany.

*Stereolithography:* The benchtop stereolithography printer Asiga Pico 2 was used for printing, postcuring of the printed parts was done with the ultraviolet radiation lamp Asiga Flash DR-301C (both Asiga, Alexandria, Australia). Single layer thickness was 50 µm and an overcuring of 125 µm was adjusted to avoid delamination of the individual layers during the printing process. Single layer thickness of 50 µm was chosen for giving good printing results at adequate printing speeds. The overcure of 125 µm was necessary to prevent the parts from delaminating during printing. Overcuring does not cause the channel structures to be blocked because the Asiga Slicing Software compensates for the overcuring when void or free hanging structures are printed. Compensation is achieved by the so-called z-compensation, which adds the overcure thickness to the size of the channel void (see Supplementary Figure S1 for details). The spectrum of the blue LED was measured using a BTC112E Fibre Coupled TE Cooled Linear CCD array spectrometer (B&W Tek, Newark, DE, USA). UV-Vis measurements were performed on an Evolution 201 UV-Vis spectrophotometer (Thermo Fisher, Waltham, MA, USA). Quartz Suprasil high precision cells were purchased from Hellma, Müllheim, Germany. HybriWell chambers were purchased from Grace Bio-Labs, Bend, OR, USA. Layer thicknesses were measured using a MT 60 M length gauge (Heidenhain, Traunreut, Germany). A Stemi 508 microscope (Zeiss, Oberkochen, Germany) with an AxioCam ERc 5s was used for microscopy.

*LED spectrum measurements:* The spectrum of the blue LED of the Asiga Pico 2 was measured using a BTC112E spectrometer. A background spectrum of the surrounding area was recorded, then the printer LED light was switched on and the spectrometer was held above the optical setup, which was close to the printing focus of the (removed) build tray. Spectra were recorded with the BWSpec software (version 4.03_23_C, B&W Tek, Newark, DE, USA). Spectra have been normalized to the maximum value.

*UV-Vis measurements:* Initiators were weighed into microcentrifuge tubes, dissolved in acetone and diluted to a concentration of 12.5 mg/mL. The solutions were transferred to a quartz glass Suprasil high precision cell and measured in an UV-Vis spectrophotometer against a blank measurement of pure acetone. Absorbers were weighed in microcentrifuge tubes, dissolved in acetone, and were diluted to a concentration of 3.76 mg/mL. The SOG solution was further diluted to a concentration of 0.12 mg/mL.

*Preparation of printing mixtures:* Stock solutions of the initiator TPO or PPO and acetone were prepared in a vortex mixer of type Reax Top (Heidolph, Schwabach, Germany). The required amount of absorber SOG, T326, or T384-2 was added in a glass vial and the initiator/acetone stock solution was added up to their respective concentrations. After mixing with the vortex mixer, the PFPE Fluorolink MD700 was added to this solution up to the final concentrations listed in Table 1. After agitating the glass vial, entrapped air bubbles in the solution where removed by using an ultrasonic bath Sonorex Da 300 (Bandelin electronic, Berlin, Germany) for 2 min at 25 °C.

*Preparation of poly(dimethylsiloxane) (PDMS):* Elastosil RT 601 A and B were mixed in a ratio of 9:1 wt %. Entrapped air bubbles were removed using a desiccator and a vacuum pump. The material was polymerized at a temperature of 70 °C for 30 min.

*Determination of optimal printing parameters*: To determine the layer thicknesses in dependence of the exposure time under the LED of the printer, the printing mixtures were pipetted into a rectangular cutout of a PDMS form to a height of 2 mm and were placed above a circular light spot of the printer with a diameter of approximately 3 mm and were exposed to the light for time periods between 4 s and 20 s. The polymer layer thicknesses were measured at three different spots using a length gauge. The results were plotted and analyzed for the slope of the resulting plots. The light intensity was set to 8.8 mW·cm^−2^.

*Solvent compatibility:* Solvent compatibility of the printed PFPE material was determined using 8 mm × 8 mm × 3 mm printed blocks of mix 3 and 5. The blocks were weighted three times and consecutively immersed in eight solvents (water, MeOH, DCM, DMF, THF, toluene, acetone, and *n*-heptane—approximately 5 mL each) for 24 h. The blocks were retrieved from the solvents, the solvent on the outside was quickly dabbed away and the blocks were immediately placed on the balance and the initial weight was recorded. The block was then re-immersed in the solvent and after weighting of the next eight blocks, the block was measured again. In this way, the weight was determined a total of three times.

*Contact angle measurement:* Contact angles were measured with an OCA15 Pro (Data Physics, San Jose, CA, USA). Static contact angles were measured using 5 µL water droplets. The surface energy was calculated using the Owens, Wendt, Rabel, and Kaelble (OWRK) method. 5 µL of diiodomethan and water were used as the nonpolar and polar testing liquid, respectively.

## 3. Results and Discussion

Several printing mixtures were used to produce PFPE microfluidic devices. To determine the optimum mixture, spectra of initiators and absorbers were recorded, mixtures were prepared and the optimum printing parameters were tested. Microfluidic devices were printed and tested for their performance. The solvent compatibility and the spectra of the printed materials were characterized.

### 3.1. UV-Vis Spectra of Initiators and Absorbers

**Figure 1:**
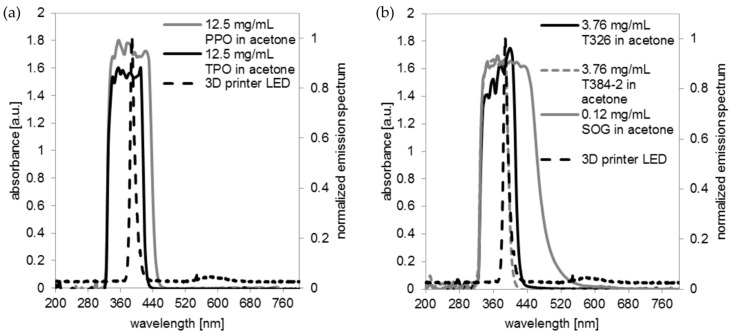
Absorption spectra of initiators and absorbers tested in comparison to the spectrum emitted by the UV LED of the Asiga Pico 2 printer (three-dimensional (3D) printer LED): (**a**) Absorption spectrum of the initiators phenylbis(2,4,6-trimethylbenzoyl)phosphine oxide (PPO) and Diphenyl(2,4,6-trimethylbenzoyl)phosphine oxide (TPO). TPO (325–415 nm) shows a less broad absorption spectrum than PPO (325–465 nm); (**b**) Absorption spectra of the absorbers tested: Tinuvin 326 (T326), Tinuvin 384-2 (T384-2), and Sudan Orange G (SOG).

In order to obtain embedded microchannels with a height of less than 1 mm, a printing formulation requires an effective initiator and a significant amount of absorber, thus ensuring that the light does not penetrate too deep into the material which would lead to undesired polymerization in layers below the lowermost layer facing the light source. The absorber must cover the whole spectral range of the light source. The UV LED of the Asiga Pico 2 emits light at 370–430 nm (see Figure 1). Two phosphine based initiators, PPO and TPO were tested for their absorption properties. At the relevant concentrations for 3D printing, PPO effectively absorbs light between 325–465 nm, while TPO shows a less broad absorption spectrum between 325 nm–415 nm (see Figure 1a). Both initiators absorb within the spectrum of the UV LED of the Asiga Pico 2 printer and were suitable for the printing process. Three absorbers, T326, T384-2, and SOG were tested for their absorption properties (Figure 1b). The absorption spectrum of T326 is well suited for printing with the UV light source, but the powder could only be mixed at a significant amount with MD700 if dissolved in acetone first (mix 1). However, this renders the printing formulation unstable for storage due to the volatility of acetone. T384-2 could also only be mixed with MD700 if being dissolved in acetone first and additionally didn’t cover the whole range of the UV LED. SOG is a very effective absorber at wavelengths between 325 nm and 560 nm. In addition, it was readily dissolvable in MD700 and the formulation was stable for storage.

### 3.2. Optimum Printing Parameters

**Figure 2:**
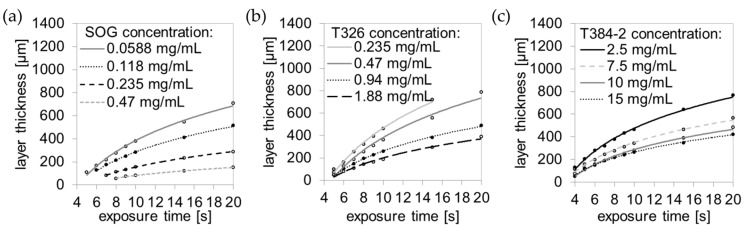
Layer thickness of printing formulations as a function of the exposure time. The polymerization curve flattens with increasing absorber concentration (final absorber concentration in MD700). Layer thickness as a function of the exposure time for different SOG (**a**), T326 (**b**), and T384-2 (**c**) concentrations.

The aim of this work was to print microfluidic channels with a layer thickness of 50 µm. To prevent the layers from delaminating during the printing process, an additional overcuring of 125 µm was required. The printing parameters for the different mixtures were optimized by analyzing the measured polymerization thickness in dependence of the exposure time (see Figure 2). The optimum printing curve should have a small slope (when plotted on a lin-log semi-logarithmic graph) to achieve most accurate printing results, but should allow for the printing of layers with a thickness of 175 µm (50 + 125 µm) within reasonable time [25]. The SOG absorber shows an optimum slope for a concentration of 0.235 mg SOG per mL of MD700, which affords a total layer thickness of 175 µm after an exposure time of 11.250 s (see Figure 2a). The T326 formulations (Figure 2b) show this optimum slope for 1.88 mg T326 per mL of MD700 (9.5 s for 175 µm layer thickness) and the T384-2 formulations at 10 mg T384-2 per mL of MD700 (6.5 s for 175 µm thickness, Figure 2c). As discussed above, these high amounts of absorbers (both T326 and T384-2) were not readily soluble in the final formulations and thus required acetone as a solvent, thereby impairing storage stability due to the volatility of acetone. Additionally, these mixtures led to a decrease of the optical transparency of the printed parts (see characterization in Section 3.5). Therefore, SOG was chosen as suitable absorber due to the fact that low concentrations are sufficient to obtain adequate light absorption and high transparency of the final parts. In the course of this work, mix 3 with an SOG concentration of 0.235 mg per mL of MD700 was used for printing the embedded microfluidic channels.

### 3.3. Printing Embedded Microfluidic Chips

**Figure 3:**
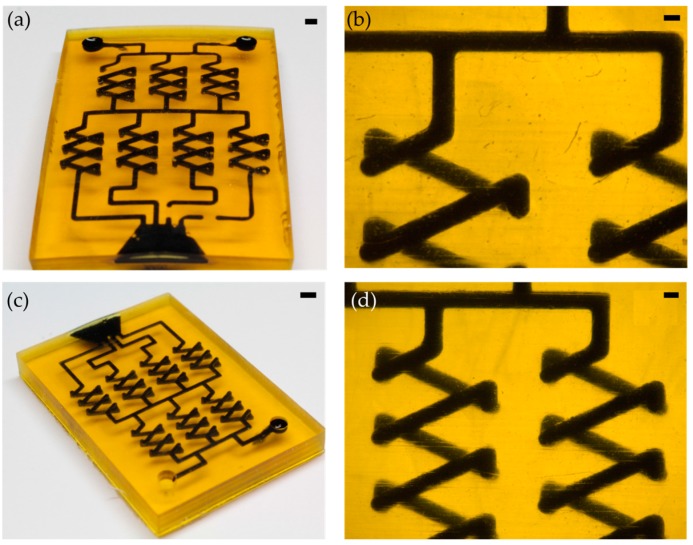
3D printed perfluoropolyether (PFPE) microfluidic chips: (**a**) Front view of a gradient generator with a channel width and height of 800 µm, filled with black printing ink (scale bar: 2 mm); (**b**) Photomicrograph of the 800 µm gradient generator (scale bar: 500 µm); (**c**) Isometric view of the 600 µm gradient generator, filled with black printing ink (scale bar: 2 mm); and, (**d**) Photomicrograph of the 600 µm gradient generator (scale bar: 500 µm).

Two exemplary microfluidic gradient generators were printed with the formulation using SOG as absorber (mix 3, see Table 1). The chips were printed with a channel width and height of 800 µm and 600 µm, respectively, with a single layer thickness of 50 µm (Figure 3a–d). After printing, the microfluidic chips were washed out with 2-propanol in an ultrasonic bath for 15 min at a temperature of 45 °C. Afterwards, the printed parts were postcured for 15 min. The microfluidic chips were filled with black ink to highlight the 3D channel networks.

**Figure 4:**
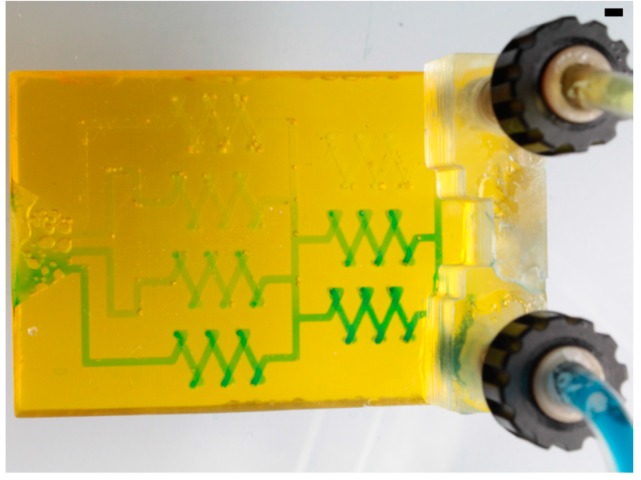
Gradient generation in a 3D printed PFPE microfluidic chip with a channel height and width of 800 µm. The upper entrance is filled with a yellow ink, the lower entrance is filled with a blue ink (scale bar: 2 mm).

In Figure 4, the microfluidic gradient generator with a channel width and height of 800 µm was filled with a blue printing ink at the upper entrance and a yellow printing ink at the lower entrance to demonstrate the gradient generation along the mixer cascade. See Supplementary Information in Figure S2 for image analysis of the RGB red channel pixel brightness evaluation.

### 3.4. Characterization of Printed Microfluidic Channels

**Figure 5:**
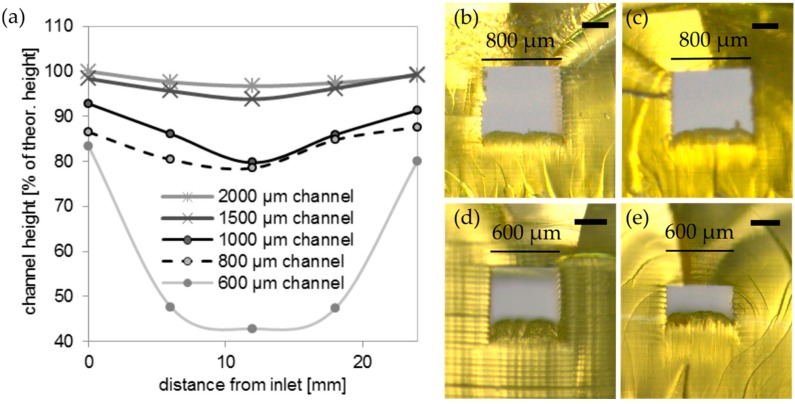
Characterization of the channel height of the printed PFPE methacrylates: (**a**) Height of a printed rectangular PFPE methacrylate channel for different spots from the inlet to the outlet. Channel heights decrease with distance from the inlet/outlet; (**b**) Lateral view of the 800 µm channel at the inlet, channel width of 800 µm, height of 692 µm (scale bar: 250 µm); (**c**) Cross section of the 800 µm channel in the middle, channel width of 800 µm, height of 628 µm (scale bar: 250 µm); (**d**) Lateral view of the 600 µm channel at the inlet, channel width of 600 µm, height of 500 µm (scale bar: 250 µm); and, (**e**) Cross section of the 600 µm channel in the middle, channel width of 600 µm, height of 257 µm (scale bar: 250 µm).

We measured the channel height and width of the printed channel to characterize the homogeneity of the printed microfluidic chips. The channel height and width were measured on printed channels with a length of 24 mm at five different spots (see Figure 5a). The highest reduction of the channel height was measured in the middle of the channels at 12 mm from the inlet. For the 1000 µm and the 800 µm channel, a reduction of 20.3% and 21.5% of the theoretical channel height was measured at 12 mm from the inlet. The highest reduction of the channel height was measured for the 600 µm channel, with a reduction of 57.2%. The cross sections of the 800 µm and the 600 µm channel at the inlet and for the middle spot are shown in Figure 5b–e.

The minimum channel dimensions of 600 µm that could be printed with the developed MD700 formulations are comparable to minimum channel sizes of around 500 µm, which have been described in literature for printing microfluidic chips using benchtop stereolithography printers **[26]**. The minimum channel dimensions in this work were mainly limited by the high viscosity (850 mPas, according to the manufacturer’s information) of the commercially available PFPE methacrylate MD700. These highly viscous resins are difficult to fully wash out of the printed microfluidic chips especially for long channels. Developing resin formulations with lower viscosities by using fluorinated acrylates with lower molecular weight and lower viscosity could help in solving this problem. Adding an appropriate solvent, like 1-butanol, to the resin formulation [27] could be another strategy to reduce the viscosity of the resin formulation with the additional advantage that the shrinkage of the material during solvent evaporation could further reduce the minimum channel dimensions.

**Figure 6:**
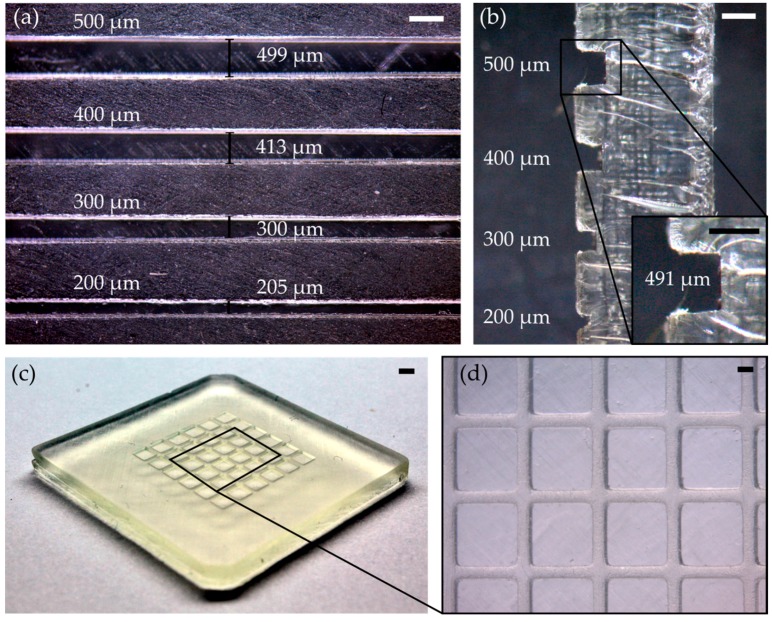
PFPE-printed open channels with a height and width of 500 µm, 400 µm, 300 µm, 200 µm and an exemplary printed PFPE chessboard structure: (**a**) The top view of the open channels shows a high conformity of the channel width (scale bar: 500 µm); (**b**) The lateral view shows the accordance of the channel heights with the set values. The inset shows the cross section of the 500 µm channel with a channel height of approximately 491 µm (scale bars: 500 µm); (**c**) Exemplary printed PFPE methacrylate chessboard structure (scale bar: 2 mm); and, (**d**) Microscope image of the chessboard from (**c**) (scale bar: 500 µm).

The formulation for printing open-channel structures or replication masters (mix 4) was identical to mix 3 for printing embedded microfluidic chips with the sole exception that no absorber was required. Rectangular open channels with a height and width from 500 µm to 200 µm were printed. As can be seen in Figure 6a, the channels show a high uniformity of the width along the channel length. The width was measured at three different spots: The 500 µm channel possesses a width of 499 ± 14 µm, the 400 µm channel a width of 413 ± 14 µm, the 300 µm channel a width of 300 ± 13 µm, and the 200 µm channel a width of 205 ± 22 µm. Figure 6b shows the cross section of the printed open channels and demonstrates the high accuracy of the printed channel height. The 500 µm channel possess a height of 491 ± 6 µm, the 400 µm channel a height of 403 ± 8 µm, the 300 µm channel a height of 307 ± 6 µm, and the 200 µm channel a height of 209 ± 7 µm. Figure 6c,d shows an exemplary printed PFPE methacrylate structure with a 500 µm chess board structure.

### 3.5. Characterization of Printed PFPE Methacrylates

**Figure 7:**
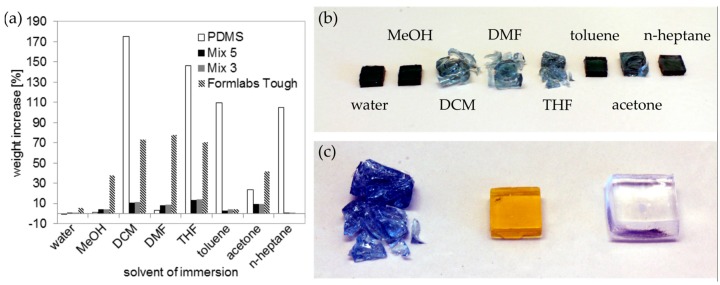
Solvent compatibility of printed PFPE methacrylates compared to poly(dimethylsiloxane) (PDMS) and the printed commercial stereolithography resin Formlabs Tough: (**a**) Rectangular 8 × 8 × 3 mm^3^ blocks were immersed in eight different solvents and the weight increase was measured after 24 h. The PFPE blocks show a lower weight increase than the PDMS blocks and the Formlabs Tough resin, which were immersed in the solvents dichloromethane (DCM), tetrahydrofuran (THF), toluene, acetone, and *n*-heptane. Mix 5 (initiator: PPO, absorber: T326) and mix 3 (initiator: TPO, absorber: SOG) show a similar swelling, which demonstrates that the absorbers and initiators have no major impact on the swelling of the printed PFPE parts; (**b**) Polymerized Formlabs Tough resin after immersion in the respective solvents for 24 h. The parts were strongly damaged by DCM, dimethylformamide (DMF), THF, and acetone; (**c**) Photograph of a Formlabs Tough block (left) and a PFPE methacrylate block (mix 3, middle) and PDMS (right) after 24 h immersion in THF.

Solvent compatibility of printed PFPE methacrylates of mix 3 and 5 were tested and compared to replicated PDMS components and the printed commercial stereolithography resin Formlabs Tough. Therefore, blocks (8 × 8 × 3 mm^3^) were immersed in the following solvents: water, MeOH, DCM, DMF, THF, toluene, acetone, and *n*-heptane. As can be seen in Figure 7, the printed PFPE methacrylates show a higher solvent compatibility than the printed commercial resin and the replicated PDMS blocks. The printed PFPE methacryates show a maximum swelling of around 14% in THF and 11% in DCM. In comparison, PDMS showed a swelling of 146% in THF and 175% in DCM. The printed Formlabs Tough parts showed the highest swelling of around 78% in DCM and 76% in DMF. However, the printed Formlabs Tough parts that were immersed in DCM, DMF, THF, and acetone were strongly damaged by the solvents (see Figure 7b,c). We further tested if the used absorbers and initiators affect the chemical resistance of the printed PFPE methacrylates. For this we compared blocks made from mix 5 (initiator: PPO, absorber: T326) and from mix 3 (initiator: TPO, absorber: SOG). As can be seen from Figure 7, the printed polymers from both mixtures show a similar swelling, suggesting that the absorbers and initiators do not have major impacts on the swelling of the printed PFPE parts. We also determined the surface energies of the printed PFPE methacrylates, which were measured at 19.5 ± 0.5 mN/m. Given this relatively low value, the printed PFPE methacrylates showed a high water contact angle of 107 ± 0.8°, which is comparable to the values described in literature [21]. Although the contact angle for water on MD700 is relatively high, at 107° the capillary pressure (calculated using Laplace-Young equation [28]) is around −1.4 mbar for a printed channel with a circular cross-section of 600 µm. Therefore, the channels do not oppose microfluidic flow in a significant manner.

**Figure 8:**
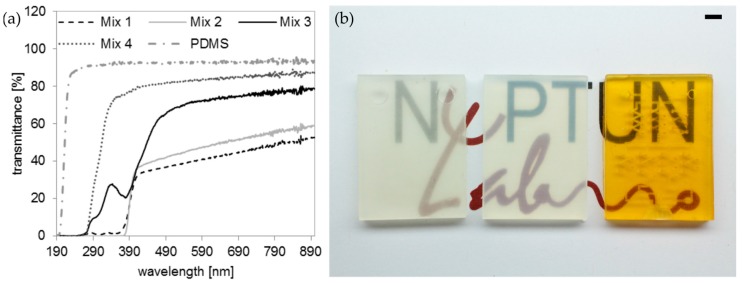
UV-Vis spectroscopy of PDMS and the printed PFPE methacrylates from mix 1, 2, 3 and 4 show different transparencies between 190–900 nm: (**a**) Transmission spectra of polymerized PDMS and PFPE methacrylates from mixtures 1, 2, 3 and 4 (thickness: 250 µm). Mix 1 shows a transparency of about 30% between 400–900 nm, mix 2 a transparency above 35% between 400–900 nm and mix 3 has a transmission above 70% between 520–900 nm. Mix 4 has a transparency of over 75% between 350–900 nm. Peaks at 330 and 280 nm are measured for the formulations prepared with TPO as photoinitiator. For comparison the UV-Vis spectrum of PDMS is shown; (**b**) Three microfluidic chips printed from mixtures 1, 2, and 3 (from left to right) show different transparencies as a function of the used absorber (scale bar: 4 mm).

We further characterized the transmittance of the printed PFPE methacrylates using UV-Vis spectroscopy. The transmittance of the printed PFPE methacrylates was further compared to polymerized PDMS parts. The transmission spectra of polymerized PFPE sheets with a thickness of 250 µm are shown in Figure 8a. Mix 1 with the absorber T326 shows a transparency above 30% between 400–900 nm, mix 2 (T384-2) a transparency above 35% between 400–900 nm. Mix 3 (SOG) shows a transmittance of over 70% between 520–900 nm. Mix 4 without any absorber shows the highest transparency above 75% between 350–900 nm. Mix 1 and mix 3 show a peak at about 330 nm and 280 nm in the UV-range, which is most likely an artefact from the photoinitiator TPO. Figure 8b shows two blocks printed from mix 1 and 2 and a microfluidic mixer that is printed from mix 3 (from left to right, thickness: 3.5 mm) placed on top of the logo of our lab (NeptunLab, Eggenstein-Leopoldshafen, Germany) showing the varying transparencies of the printed PFPE parts. As mentioned above, the high concentration of T326 and T384-2 required for printing microfluidic channels below 1 mm is not readily soluble in the formulation and required a prior dissolving in acetone. Consequently, a decrease of the transparency of the printed PFPE methacrylates was observed. SOG, which possesses a high light absorption at low concentrations, is readily soluble in the mixture and the printed parts have a higher optical clarity.

## 4. Conclusions

In this work, we have demonstrated a new method to fabricate microfluidic chips in highly fluorinated PFPE methacrylates using stereolithography 3D printing. We have developed material formulations that can be printed in commercial stereolithography printers. We have shown that by adjusting the amount of initiator and absorber transparent microfluidic chips can be printed. The printed chips show high chemical resistance in the tested organic solvents. We believe that printing highly fluorinated PFPE methacrylates will find numerous applications from chemical resistant microfluidic valves to chemistry-on-chip applications.

## Figures and Tables

**Table 1:** Composition of printing formulations mix 1–5.

	Mix 1	Mix 2	Mix 3	Mix 4	Mix 5
Initiator (mg/mL) ^1^	6.25 TPO	9 PPO	6.25 TPO	6.25 TPO	9 PPO
Absorber (mg/mL) ^1^	1.88 T326	10 T384-2	0.235 SOG	-	0.5 T326

^1^ Final concentration in MD700.

## Supplementary Materials

The following materials are available online at http://www.mdpi.com/2072-666X/9/3/115/s1, Figure S1: Overcuring and z-compensation of the Asiga Pico 2 printer: (a) Initial CAD file; (b) Slicing of the CAD file without the overcuring effect. The sliced CAD file has the same dimension like the initial CAD file; (c) Overcuring (l+o) is needed to prevent the slices from delamination during the printing process; (d) During the slicing process the overcuring is taken into account by the so called z-compensation. The shown sliced channel structure is higher than the initial CAD file; (e) The difference is polymerized by the overcuring during the printing process. The printed channel has the height of the initial CAD file. Figure S2: Determination of the color intensity in a PFPE-printed microfluidic channel (800 µm channel height and width) at different mixing positions: (a) The color intensity ((red+green+blue)/3) at different measurement spots shows the gradient generation in the microfluidic chip; (b) Greyscale image of the microfluidic chip showing the position of the measurement spots.
