# Supplementary material for: Highly Fluorinated Methacrylates for Optical 3D Printing of Microfluidic Devices
Source: Micromachines (Basel). 2018 Mar 8;9(3):115. doi: 10.3390/mi9030115 (PMC6187856; doi:10.3390/mi9030115)
Supplement: Supplementary file 1 [file micromachines-09-00115-s001.pdf]

Supplementary Materials: Highly Fluorinated Methacrylates for Optical 3D Printing of Microfluidic Devices

Frederik Kotz, Patrick Risch, Dorothea Helmer and Bastian E. Rapp

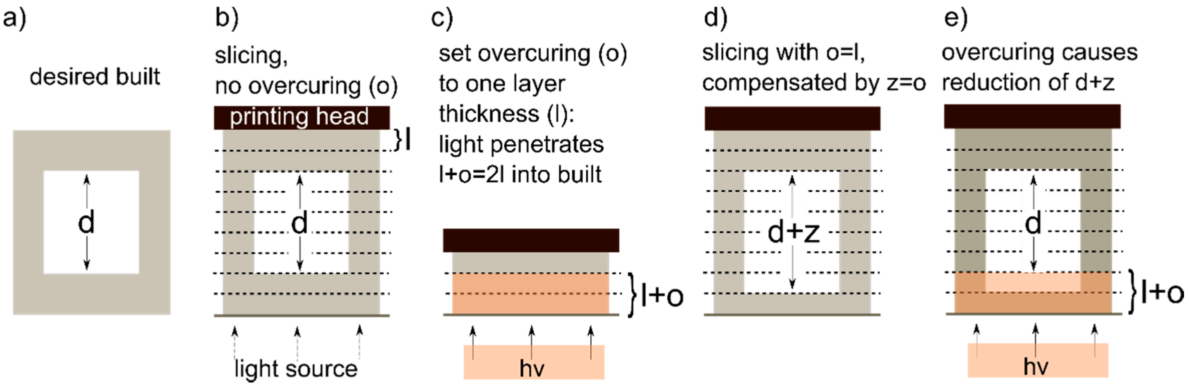

Figure S1. Overcuring and z-compensation of the Asiga Pico 2 printer: (**a**) Initial CAD file; (**b**) Slicing of the CAD file without the overcuring effect. The sliced CAD file has the same dimension like the initial CAD file; (**c**) Overcuring (l+o) is needed to prevent the slices from delamination during the printing process; (**d**) During the slicing process the overcuring is taken into account by the so called z-compensation. The shown sliced channel structure is higher than the initial CAD file; (**e**) The difference is polymerized by the overcuring during the printing process. The printed channel has the height of the initial CAD file.

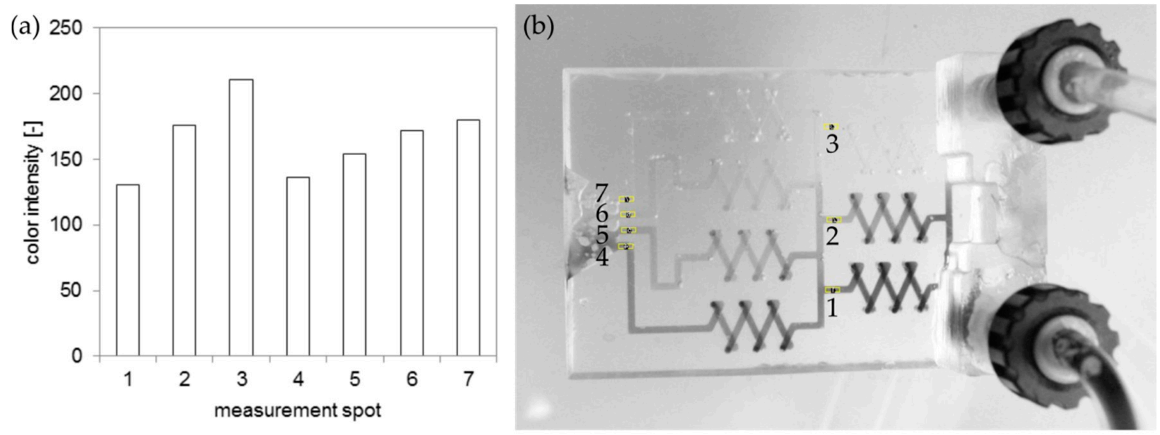

Figure S2. Determination of the color intensity in a PFPE-printed microfluidic channel (800 µm channel height and width) at different mixing positions: (**a**) The color intensity ((red+green+blue)/3) at different measurement spots shows the gradient generation in the microfluidic chip; (**b**) Greyscale image of the microfluidic chip showing the position of the measurement spots.
